# Reduced left atrial contractile strain with speckle tracking analysis predicts abnormal plasma NTproBNP in an asymptomatic community population

**DOI:** 10.1186/s12947-022-00297-y

**Published:** 2022-11-26

**Authors:** Lin Liu, Baowei Zhang, Ying Yang, Litong Qi, Shuo Wang, Lei Meng, Wei Ma, Yong Huo

**Affiliations:** 1 Department of Cardiology, Central Laboratory of Echocardiography, Peking University First Hospital, No. 1, Dahongluochang Street, West District, Beijing, 100034 People’s Republic of China; 2 Department of Cardiology, Peking University Shougang Hospital, Beijing, China; 3 Beijing Hypertension League Institute, Beijing, China

**Keywords:** Left atrium, Strain, Strain rate, Left atrial stiffness index, NTproBNP, Speckle-tracking echocardiography

## Abstract

**Background:**

The left atrium (LA) is closely related to left ventricular diastolic function. Two-dimensional speckle tracking strain and strain rate (SR) imaging has been applied in the study of LA function. We intended to explore the relationship between global LA deformation parameters and plasma NTproBNP levels in asymptomatic community residents with normal ejection fraction and normal LA volume.

**Methods:**

A cross-sectional sample of Beijing residents underwent comprehensive Doppler echocardiography and medical record review in 2009. Global LA longitudinal strain and SR indexes were obtained in the apical four-chamber view. LA stiffness index (LASI) was calculated as the ratio of early diastolic velocity of transmitral flow/early diastolic mitral annular motion velocity (E/E') to LA reservoir strain.

**Results:**

A total of 620 individuals (mean age = 65.8 years, left ventricular ejection fraction = 70.8%, LA volume index = 17.9 ml/m^2^) were investigated in our study. 117 individuals had increased plasma NTproBNP (≥ 125 pg/ml). LA reservoir and contractile function by LA strain and SR indexes were significantly reduced in the abnormal NTproBNP group compared with the normal NTproBNP group. Multiple regression analysis indicated that LA contractile strain was a negative predictor of plasma NTproBNP in addition to indexed LA volume and E/E'. LASI was higher in the abnormal NTproBNP group and was significantly correlated with NTproBNP (*r* = 0.342, *P* < 0.001). The area under ROC analysis for LASI in predicting elevated plasma NTproBNP was 0.690, similar with LA contractile strain, E/E’ and LAVI. The cut-off value of LASI was 0.612.

**Conclusions:**

LA reservoir and contractile functions demonstrated by LA strain and SR were significantly impaired in the community-based population with increased plasma NTproBNP levels. LA contractile strain adds incremental information in predicting abnormal NTproBNP levels. As a single index, LASI showed similar diagnostic value with LAVI and E/E’ in predicting abnormal NTproBNP.

## Introduction

Left atrial (LA) function is a useful barometer of LV diastolic function and vital for overall cardiac performance. LA mechanical function includes reservoir, conduit and pump function which contribute to left ventricular filling at different stages of the cardiac cycle [1]. Two-dimensional speckle tracking strain and strain rate(SR) imaging has been proposed as a new tool to evaluate LA function with considerable feasibility and reproducibility [2]. LA reservoir strain can predict elevated LV filling pressures [3, 4], classify left ventricular diastolic dysfunction [5, 6], discriminate heart failure with preserved ejection fraction (HFpEF) more accurately than conventional echocardiographic measures or the guidelines algorithm [7, 8], and is also associated with the prognosis in patients with HFpEF [9, 10].

N-terminal pro-brain natriuretic peptide (NTproBNP), secreted mainly by the ventricles in case of volume expansion and pressure overload, is a noninvasive marker of elevated LV filling pressure, and is regarded as an important diagnostic and prognostic tool in patients with heart failure [11]. The upper limit of normal plasma NT-proBNP is 125 pg/ml in the non-acute setting according to the 2016 ESC heart failure guideline [12]. Plasma NTproBNP was once regarded as a suboptimal screening test for preclinical ventricular dysfunction in community-based populations [13], but evidence from a meta-analysis of 40 prospective studies also supports the potential role of NTproBNP in the assessment of cardiovascular risk in general populations [14].

We investigated the relevance of LA deformation parameters assessed by two-dimensional speckle tracking imaging with plasma NTproBNP levels in a community-based population with normal left ventricular ejection fraction (LVEF) and normal LA volume. We speculate that LA function by LA strain and SR indexes in people with increased NTproBNP might be different from those with normal NTproBNP. We aim to determine the role of LA deformation parameters in predicting plasma NTproBNP levels, and to assess which of the indexes, separately or in combination, is a better correlate.

## Methods

### Population

**Fig. 1:**
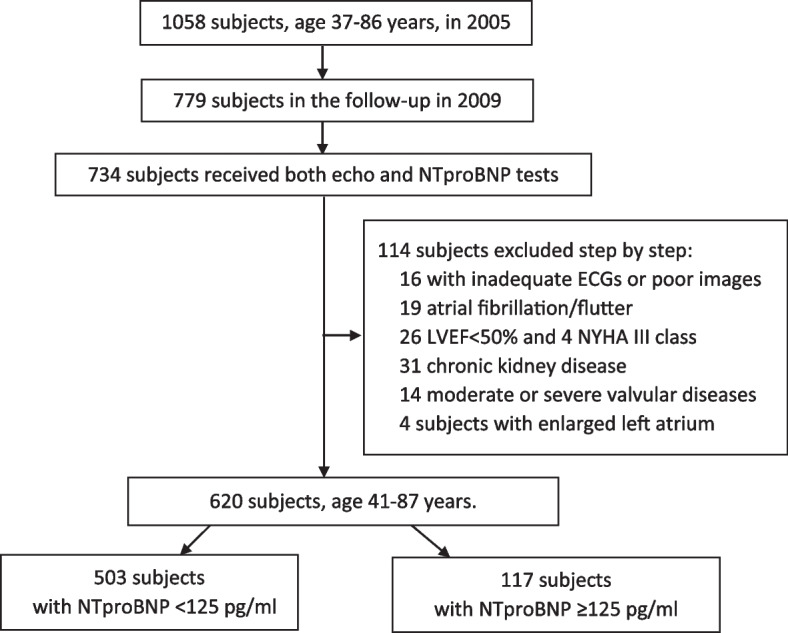
Flow chart of the study population

Our study enrolled the cohort in the community of the Capital Steel Corporation set up by Beijing Hypertension League Institute, including 1058 subjects, aged between 37–86 years old in 2005 [15]. Among them, 779 subjects took part in the follow-up in 2009. Clinical characteristics, echocardiographic examinations, and fasting samples were collected. The cross-sectional data of 734 subjects with full records were identified. Of these, 16 subjects were excluded for inadequate electrocardiograms or poor imaging quality. 19 subjects were excluded for history of atrial fibrillation or flutter. 26 subjects had reduced LVEF (< 50%) and 4 subjects with NYHA class III were excluded. 31 chronic kidney disease subjects with an estimated glomerular filtration rate (eGFR) < 60 ml/min.1.73m^2^ and 14 subjects with moderate or severe valvular diseases were excluded. Then, 4 subjects with enlarged LA (LA volume index > 34 ml/m^2^) were excluded to avoid enlarged LA as a confounding factor on LA phasic function. The final study consisted of 620 individuals (Fig. 1 flow chart). The study was in compliance with the Declaration of Helsinki and approved by the Ethics Committee of Peking University First Hospital. All subjects gave their written informed consent for participation.

### Assessment of clinical parameters

Cardiovascular diseases and risk factors were confirmed based on a review of data collected from hospitalizations and outpatient records. Fasting blood samples were collected for analysis using standard techniques. eGFR was calculated using modified MDRD equations based on Chinese patients [16]. NTproBNP was tested by eletrochemiluminescence immunoassay (Elecsys, Roche Diagnostics, Germany). According to the 2016 ESC heart failure guideline, the upper limit of normal plasma NTproBNP in the non-acute setting is 125 pg/ml, which suggests that patients with normal NTproBNP concentrations are unlikely to have heart failure [12]. Tests of biochemical indexes were completed in the clinical laboratory of Peking University First Hospital, and a quality control standard (ISO 15189) was achieved.

### Standard echocardiography

Echocardiographic examinations were performed using a Vivid 7 ultrasound system (GE Healthcare, Horten, Norway) equipped with a 2–4 MHz transducer with a frame rate of at least 50 frames per second according to the guidelines [17, 18]. Images in cineloop format from 3 consecutive beats were stored for a offline analysis. The maximal LA volume (LAV) was calculated using the biplane dimension-length formula: LAV (ml) = π/6 × (anteroposterior diameter) × (longitudinal diameter) × (transverse diameter) [18]. The LAV index (LAVI) was calculated as LAV/body surface area (BSA). Left ventricular mass (LVM) was calculated with the Devereux formula: LVM (g) = 0.8 × 1.04 × [(left ventricular end-diastolic internal diameter + intraventricular septal thickness + left ventricular posterior wall thickness)^3^- (left ventricular end-diastolic internal diameter)^3^] + 0.6 [18]. The LVM index (LVMI) was subsequently calculated as LVM/BSA. LVEF was assessed by modified biplane Simpson’s method. Transmitral flow velocities were obtained including peak velocities during early diastole (E) and late diastole (A). Values shown for peak early (E’) and late (A’) diastolic mitral annular velocities were averages of the values obtained at septal and lateral positions.

### Measurements of LA strain and SR

**Fig. 2:**
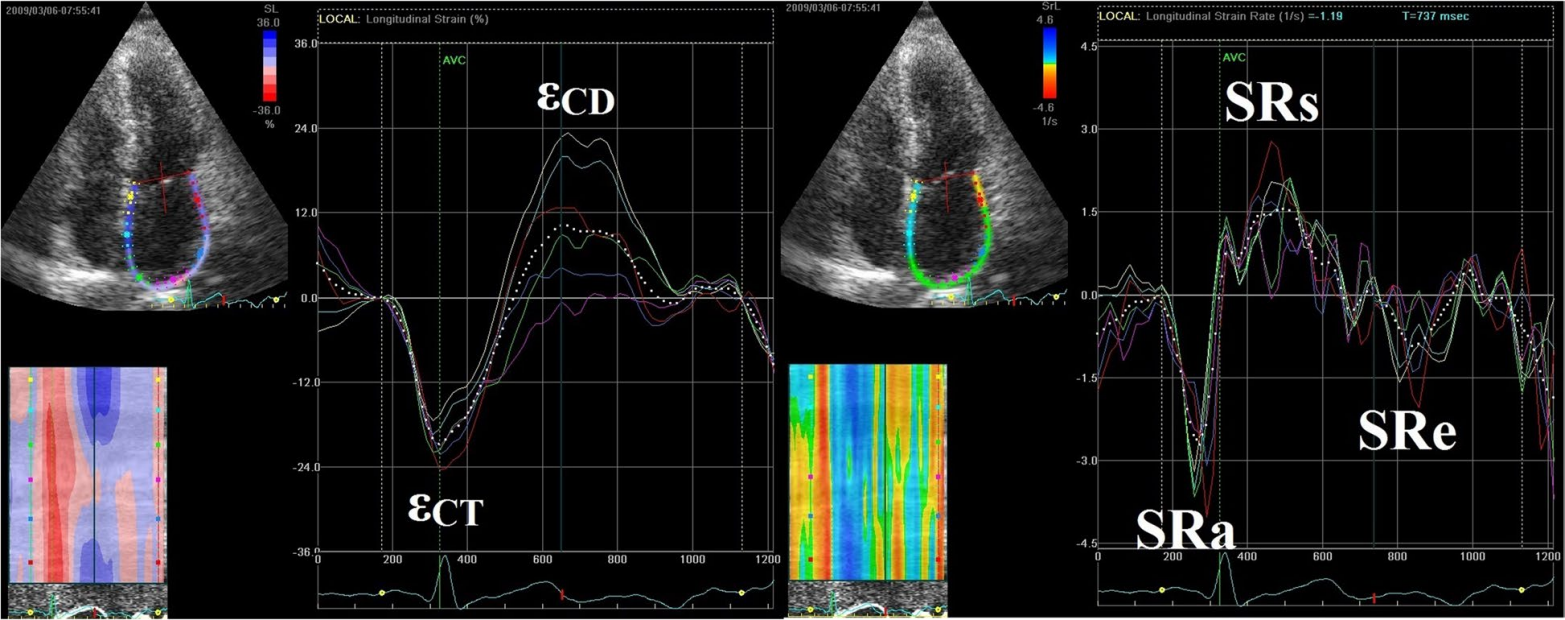
Left atrial strain and strain rate measured by speckle tracking imaging on apical four-chamber views

Strain measures the myocardial deformation during a cardiac cycle, and strain rate (SR) measures the tissue velocity gradient within the myocardium. LA strain (ε) and SR were analysed by the 2D speckle tracking technique using EchoPAC software (Version 11.0, GE Healthcare) according to Sergio Mondillo’s method [19], by two experienced investigators who were blinded to clinical and other echocardiographic characteristics of the population. The grayscale 2D images acquired in the standard 4-chamber apical views were used. The software divided the LA wall into 6 segments, lateral and septal annular, lateral and septal mid-cavity and lateral and septal rear segments. LA global longitudinal strain and SR measurements were obtained as the average values (white dotted line as shown in Fig. 2).

The zero reference for LA strain is set at the onset of the P wave. The first peak negative strain (ε_CT_) corresponds to the LA contractile function, and the following peak positive strain (ε_CD_) corresponds to the LA conduit function. ε_R_, as the sum of ε_CD_ and ε_CT_, corresponds to LA reservoir function (Fig. 2). LASI is calculated as the ratio of E/Eʹ to LA reservoir strain [20].

The LA SR pattern is characterized by a positive wave occurring during ventricular systole and two negative waves during ventricular diastole. Peak positive global SR (SRs) reflects LA reservoir function, the first peak negative SR (SRe) reflects LA conduit function, and the second peak negative SR (SRa) reflects LA contractile function [21] (Fig. 2). For negative LA strain and SR variables, absolute values were used.

### Intra-observer and inter-observer variability

Intra-observer and inter-observer variability of LA strain and SR indexes was assessed in 20 randomly selected subjects. To assess intra-observer variability, selected images were analyzed at a different time by the same observer. To assess inter-observer variability, selected images were analyzed by another observer blinded to the values.

### Statistical analysis

We analysed the differences between subjects with normal and increased plasma NTproBNP levels. Continuous variables with a normal distribution were expressed as the mean ± standard deviation (SD), and an independent t-test was used. Continuous variables with obvious skew distributions by Shapiro–Wilk analysis and histograms were expressed as medians and quartiles, and the Mann–Whitney U test was used. Categorical variables were compared using chi-square tests and Fisher’s exact tests as appropriate. Spearman correlation was used to analyse associations between echocardiographic parameters and NTproBNP. Forward conditional logistic regression was performed to explore the independent factors for the prediction of abnormal NTproBNP. Receiver operating characteristic (ROC) curves were used to determine the diagnostic performance of LA strain and SR indexes as well as other echocardiographic parameters to detect elevated NTproBNP. DeLong test was performed for comparison of ROC curves using MedCalc (Version 20.111, MedCalc Software Ltd, Ostend, Belgium). Comparisons of the intra-observer and inter-observer agreement of LA speckle-tracking parameters were assessed by Bland–Altman analysis using MedCalc (Version 20.111, MedCalc Software Ltd). Other data analysis was performed using SPSS 20.0 software (IBM-SPSS, Armonk, NY, USA). The results were considered statistically significant when the *P* value was < 0.05.

## Results

### Clinical characteristics

Clinical characteristics and echocardiographic findings are shown in Table 1. Among all subjects (age = 65.8 ± 5.9), the plasma NTproBNP level was 62.5 (32.2–105.3) pg/ml. The average LVEF was 70.8 ± 9.4%, LVMI was 91.6 ± 22.5 g/m^2^, and LAVI was 17.9 ± 4.8 ml/m^2^.

**Table 1:** Clinical characteristics and echocardiographic parameters of the study subjects

	All (*n* = 620)	NTproBNP < 125 (*n* = 503)	NTproBNP ≥ 125 (*n* = 117)
**Clinical characteristics**			
Age (years)	65.8 ± 8.9	64.6 ± 8.9	71.2 ± 6.5**^**
Female	327 (52.7%)	263(52.3%)	64(54.7%)
BMI (kg/m^2^)	25.6 ± 3.3	25.8 ± 3.3	24.6 ± 3.1**^**
smoking	200 (32.3%)	168 (33.4%)	32 (27.4%)
Hypertension	499 (80.5%)	400(79.5%)	99 (84.6%)
Diabetes	176 (28.4%)	135(26.8%)	41 (35.0%)
CAD	104 (16.8%)	72(14.3%)	32 (27.4%)**^**
History of heart failure	13 (2.1%)	8(1.6%)	5 (4.3%)
Stroke	120 (19.4%)	93(18.5%)	27 (23.1%)
Heart rate(bpm)	73.6 ± 13.2	74.1 ± 12.7	71.7 ± 14.8
Total cholesterol(mmol/L)	5.24 ± 1.06	5.28 ± 1.03	5.06 ± 1.15*
Low-density lipoprotein(mmol/L)	3.26 ± 0.88	3.29 ± 0.87	3.10 ± 0.89*
High-density lipoprotein(mmol/L)	1.32 ± 0.30	1.32 ± 0.30	1.32 ± 0.33
Triglyceride (mmol/L)	1.41 (0.98–2.01)	1.47(1.01–2.05)	1.21(0.90–1.72)**^**
Blood glucose (mmol/L)	6.57 ± 2.07	6.52 ± 1.88	6.82 ± 2.71
eGFR (ml/min.1.73m^2^)	87.2 ± 14.3	88.2 ± 14.1	82.5 ± 14.3*
Uric Acid(umol/L)	303.3 ± 76.4	305.1 ± 74.8	295.7 ± 82.6
Hs-CRP	1.18(0.50–2.76)	1.14(0.49–2.59)	1.48(0.54–3.43)
NTproBNP (pg/ml)	62.5(32.2–105.3)	48.3(28.1–77.3)	174.7(141.9–229.3) **^**
**Conventional measurements**			
LVDd (cm)	4.63 ± 0.48	4.63 ± 0.48	4.62 ± 0.49
LVEF(%)	70.8 ± 9.4	70.9 ± 9.2	70.4 ± 10.3
LVMI (g/m^2^)	91.6 ± 22.5	90.3 ± 22.4	97.0 ± 22.6**^**
LAVI (ml/m^2^)	17.9 ± 4.8	17.4 ± 4.7	20.1 ± 4.9**^**
E/A	0.85 ± 0.24	0.85 ± 0.24	0.85 ± 0.24
E'(cm/s)	7.6 ± 2.1	7.8 ± 2.1	6.9 ± 1.9**^**
A'(cm/s)	11.2 ± 1.7	11.4 ± 1.7	10.6 ± 1.7**^**
E/E’ ratio	10.8 ± 3.5	10.4 ± 3.2	12.6 ± 4.0**^**
E/E' > 14	99(16.0%)	58(11.5%)	41(35.0%)**^**
VTR (m/s)	2.46 ± 0.33	2.44 ± 0.31	2.53 ± 0.37*
VTR > 2.8 m/s	69(11.1%)	41(8.2%)	28(23.9%)**^**
**LA strain and SR**			
LA reservoir function			
ε_R_(%)	21.61 ± 5.47	22.00 ± 5.50	19.93 ± 5.00**^**
SR_s_(s^−1^)	1.09 ± 0.31	1.11 ± 0.31	0.97 ± 0.28**^**
LA conduit function			
ε_CD_ (%)	9.99 ± 4.41	10.08 ± 4.33	9.62 ± 4.73
SR_e_(s^−1^)	0.91 ± 0.50	0.91 ± 0.36	0.88 ± 0.86
LA contractile function			
ε_CT_(%)	11.62 ± 3.70	11.93 ± 3.79	10.30 ± 2.96**^**
SR_a_(s^−1^)	1.53 ± 0.53	1.58 ± 0.54	1.32 ± 0.38**^**
LASI	0.54 ± 0.25	0.51 ± 0.23	0.68 ± 0.30 **^**

Values are mean ± SD / median(quartiles) or %;*: *P* < 0.05, **^**: *P* ≤ 0.01

*BMI* Body mass index, *CAD* Coronary artery disease, *eGFR* Estimated glomerular filtration rate, *LVDd* Left ventricular diastolic diameter, *LVEF* Left ventricular ejection fraction, *LVMI* Left ventricular mass index, *LAVI* Maximal left atrial volume index, *E* Peak velocity during early diastolic of mitral flow by pulsed Doppler, *A* Peak velocity during late diastolic of mitral flow by pulsed Doppler, *E’* The average of septal and lateral mitral annular early diastolic peak velocity, *A’* The average of septal and lateral mitral annular late diastolic peak velocity, *VTR* Peak velocityof tricuspid regurgitation, available in 476 patients, *LASI* Left atrial stiffness index

The global LA ε_R_, ε_CD_ and ε_CT_ were 21.61 ± 5.47%, 9.99 ± 4.41%, and 11.62 ± 3.70%, respectively. The global LA SRs, SRe and SRa were 1.09 ± 0.31 s^−1^, 0.91 ± 0.50 s^−1^, and 1.53 ± 0.53 s^−1^, respectively.

### LA volume and deformation parameters in the abnormal NTproBNP group

Subjects were categorized into two groups by NTproBNP level: 503 subjects with NTproBNP < 125 pg/ml and 117 subjects with NTproBNP ≥ 125 pg/ml. Expected between-group differences were found in age, BMI, eGFR, the prevalence of coronary artery disease and heart failure history. Subjects with abnormal NTproBNP had higher LVMI and E/E’ ratios (*P* ≤ 0.01). No differences in LV diameters or LVEF were detected between the two groups.

Compared with the normal NTproBNP group, subjects with abnormal NTproBNP had significantly increased LAVI (20.1 ± 4.9 vs 17.4 ± 4.7 ml/m^2^, *P* ≤ 0.01), and decreased LA deformation indexes demonstrating impaired LA reservoir function (εR:19.93 ± 5.00% vs 22.00 ± 5.50%, SRs:0.97 ± 0.28/s vs 1.11 ± 0.31/s) and pump function (εCT: 10.30 ± 2.96% vs 11.93 ± 3.79%, SRa:1.32 ± 0.38/s vs 1.58 ± 0.54/s) (*P* ≤ 0.01), while LA conduit function by εCD and SRe remained similar. LASI was significantly higher in the abnormal NTproBNP group (0.68 ± 0.30 vs 0.51 ± 0.23, *P* ≤ 0.01) (Table 1).

### Relationships between LA strain/SR indexes and NTproBNP

**Fig. 3:**
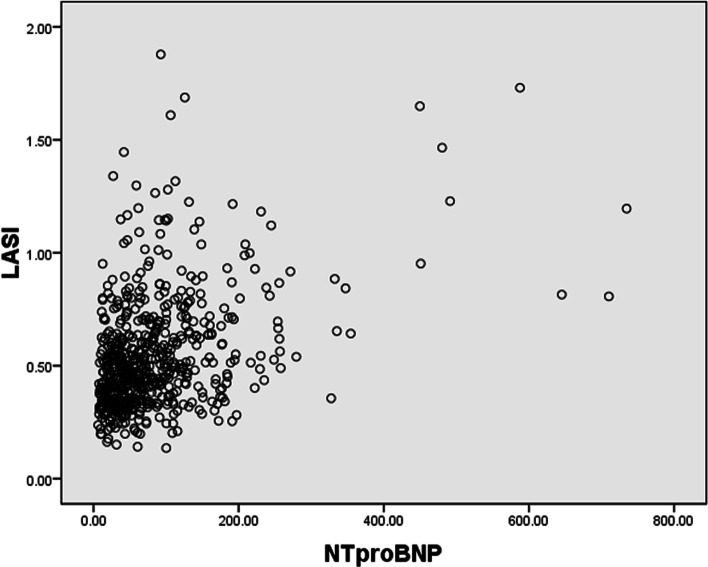
Correlations between LASI and plasma NTproBNP levels in asymptomatic community population

Spearman correlation analysis found that ε_R_ and ε_CT_ were only mildly negatively associated with plasma NTproBNP (*r* = -0.2 ~ -0.3, *P* < 0.001). LASI was significantly correlated with other echocardiographic parameters demonstrating raised left ventricular filling pressures (E', LAVI, TR velocity) and NTproBNP (Table 2). A scatter plot of LASI and NTproBNP (*r* = 0.342, *P* < 0.001) was shown in Fig. 3.

**Table 2:** Correlations between LA deformation parameters andNTproBNP &conventional echocardiographic measures, which demonstrate raised left ventricular filling pressures

	ε_R_		ε_CT_		LASI	
	r	*p* value	r	*p* value	r	*p* value
NTproBNP	-0.226	< 0.001	-0.213	< 0.001	0.342	< 0.001
E'	0.268	< 0.001	-0.071	0.077	-0.615	< 0.001
E/E'	-0.090	0.025	0.010	0.808	/	/
LAVI	-0.066	0.103	-0.139	0.001	0.177	< 0.001
VTR (m/s)	-0.105	0.022	-0.076	0.096	0.183	< 0.001

Correlation between LASI and E/e’ does not apply since E/e’ is used to calculate LASI

After fully adjusting for confounding factors, multivariate logistic regression analysis demonstrated that ε_CT_, LAVI, and E/E' > 14 were independent predictors of abnormal NTproBNP in addition to age, BMI and history of heart failure (Table 3). The odds ratio for ε_CT_ was below 1, suggesting negative impacts on plasma NTproBNP. LASI was not an independent influential factor of NTproBNP.

**Table 3:** Multivariate binary logistic regression analysis of clinical and echocardiographical variables to predict abnormal NTproBNP in the whole population

	Ratio	95.0% Confidence Interval	*p* value
ε_CT_	0.873	0.817–0.933	< 0.001
Age	1.088	1.054–1.124	< 0.001
BMI	0.868	0.806–0.935	< 0.001
History of heart failure	3.738	1.092–12.791	0.036
LAVI	1.093	1.044–1.146	< 0.001
E/E' > 14	2.899	1.687–4.983	< 0.001
constant	0.017		

Adjusted for age, sex, BMI, eGFR, hypertension, diabetes, CAD, stroke, LVMI, LAVI, E', VTR > 2.8 m/s, εR, SRs, εCD, SRe, SRa, LASI

### ROC analysis for abnormal NTproBNP

**Fig. 4:**
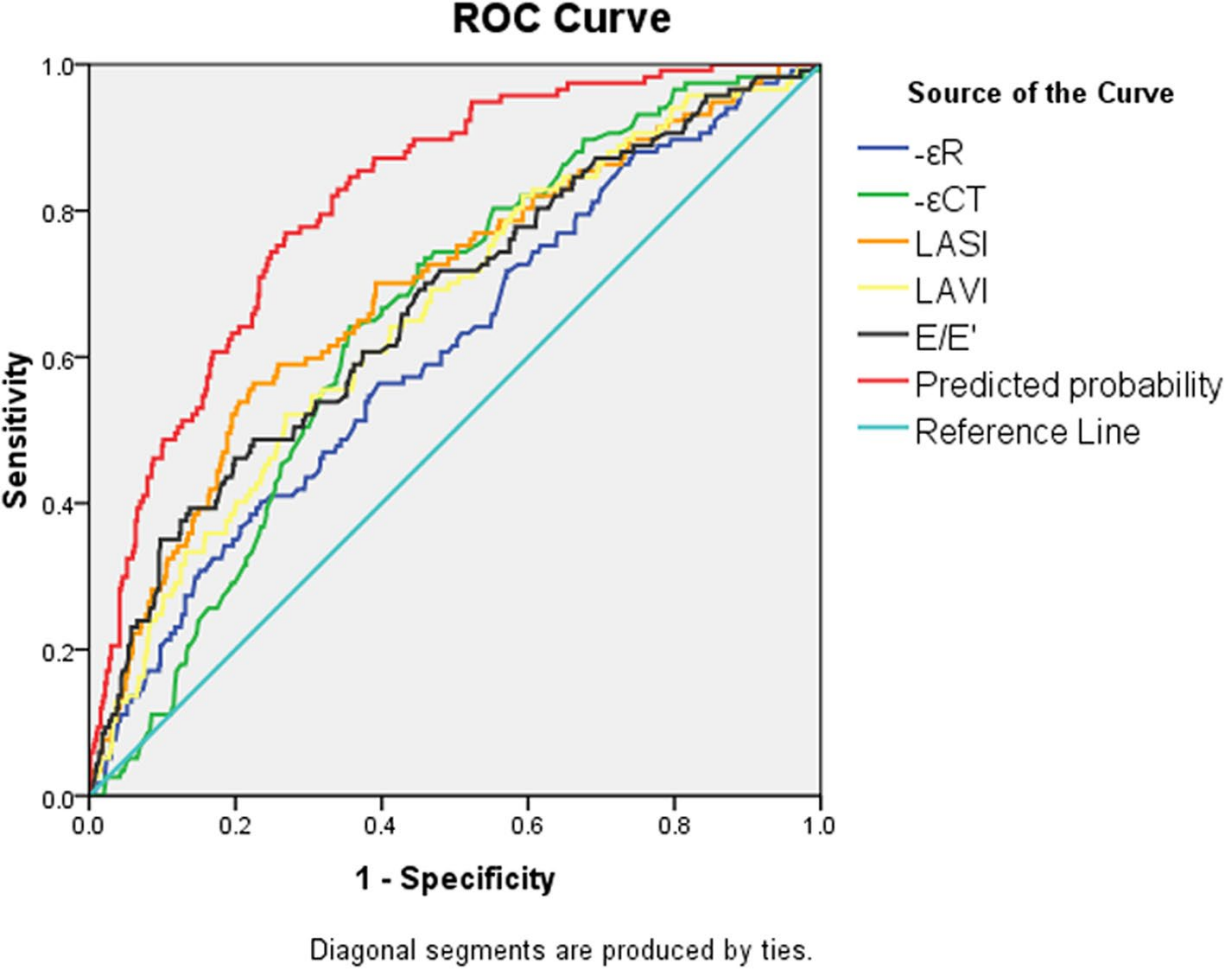
Receiver operating characteristic curve in predicting abnormal NTproBNP levels

The diagnostic performance of LASI as a single index in predicting elevated NTproBNP (≥ 125 pg/ml) (AUC 0.690, cut-off value 0.612, specificity: 0.775, sensitivity:0.564), was similar with -ε_CT_ (AUC 0.650), LAVI (AUC 0.658) and E/E' (AUC 0.667), but better than -ε_R_ (AUC 0.608) by ROC analysis and DeLong test (Table 4, Fig. 4).

**Table 4:** Receiver operating characteristic curve in predicting abnormal NTproBNP (NTproBNP ≥ 125 pg/ml) by MedCalc

	AUC	95% Confidence Interval	Cutoff point	sensitivity	specificity	*p* value
-ε_R_	0.608	0.569–0.647	-20.28	0.604	0.564	< 0.001
-ε_CT_	0.650	0.611–0.688	-10.88	0.644	0.641	< 0.001
LASI	0.690	0.652–0.726	0.612	0.775	0.564	< 0.001
E/E'	0.667	0.628–0.704	12.44	0.801	0.462	< 0.001
LAVI	0.658	0.620–0.696	19.899	0.732	0.521	< 0.001
Predicted probability	0.815	0.782–0.845	0.184	0.732	0.769	< 0.001

The ROC curve was further fitted through the predicted probability of the logistic regression model of increased NTproBNP listed in Table 3. The AUC for the regression model was 0.815 (Fig. 4), and the regresseion model was significantly better than other single echocardiographic indexes by DeLong test (*P* < 0.001), which showed that combining LA ε_CT_ and conventional echocardiographic measures (including LAVI and E/E') improved the diagnostic accuracy of abnormal BNP.

### Reproducibility of LA speckle-tracking parameters

The inter-observer and intra-observer agreements of LA strain and strain rates were shown using mean difference bias and 2-SD limits of agreement (LOA) in Table 5.

**Table 5:** The intra-observer and inter-observer agreement analysis

Parameter	Intra-observer agreement analysis		Inter-observer agreement analysis	
	Bias	95% LOA	Bias	95% LOA
ε_CT_	-0.21	-2.56–2.15	0.92	-1.93–3.78
ε_CD_	-0.48	-4.46–3.50	-0.20	-5.68–5.29
SRs	0.01	-0.32–0.35	-0.10	-0.43–0.24
SRe	-0.01	-0.28–0.26	-0.08	-0.39–0.23
SRa	-0.04	-0.41–0.33	-0.06	-0.51–0.38

*LOA* Limits of agreement

## Discussion

In this study, we explored LA function by speckle tracking analysis in community people with normal LVEF, normal LA volume and no obvious heart failure symptoms. Impaired LA reservoir and pump function were found in subjects with abnormal NTproBNP. LA contractile strain was an independent factor of plasma NTproBNP and added incremental information in predicting abnormal NTproBNP levels to that provided by LA volume and E/E' assessment. LASI was correlated with plasma NTproBNP and showed similar diagnostic performance with conventional measures (including LA contractile strain, LAVI and E/E’) in predicting elevated NTproBNP.

### Reduced LA function in community population

A meta-analysis revealed normal reference ranges for reservoir strain of 39% (95% CI, 38%-41%), for conduit strain of 23% (95% CI, 21%-25%), and for contractile strain of 17% (95% CI, 16%-19%) in healthy participants without cardiac risk factors [22]. In our study, the subjects had reduced LA strain (global LA ε_R_ 21.61 ± 5.47%, ε_CD_ 9.99 ± 4.41%, and ε_CT_ 11.62 ± 3.70%), probably due to heterogeneous characteristics and a high percentage of comorbid conditions such as hypertension (81.5%) and diabetes (28.4%). Hypertension and diabetes mellitus are both associated with morphologic and functional abnormalities of the LA. An earlier study has already shown that hypertension and diabetes are both associated with decreases in all LA strain and SR indexes [23].

Subjects in our study had a normal LA size and the average LAVI was 17.9 ± 4.8 ml/m^2^, while LA deformation mechanics were obviously impaired. It suggested that LA phasic function decreased prior to the onset of LA enlargement, which was in line with other studies involving hypertensive and diabetic patients [23–25]. LA dysfunction was associated with LA fibrosis [26], and LA strain may become a marker of LA fibrosis [27].

As acknowledged, there is a close interdependence between LV and LA function. With abnormal LV relaxation, LA conduit function decreases, while the relative contribution of LA reservoir and contractile function increases to maintain optimal LV end-diastolic volume, representing an important compensatory mechanism. However, with further progression of LV diastolic dysfunction and increased LA stiffness, the LA pump function decreases, and LA serves predominantly as a conduit [28, 29]. The progression of LA dysfunction is a key factor leading from left ventricular dysfunction to the development of heart failure [27]. In a study on women, LA reservoir and conduit function progressively decreased with increasing grades of left ventricular dysfunction (LVDD), whereas contractile function augmented in grade 1 LVDD before being reduced in patients with grade 2 LVDD [30]. Another study on hypertensive patients showed that LA reservoir and conduit function gradually decreased from enlarged LA to hypertrophic LV [24]. In our study, asymptomatic community subjects with abnormal NTproBNP had worsened LA reservoir and contractile function. These discrepancies could be due to distinct pathophysiological stages in patients with different diseases.

### Relationship between LA strain and NTproBNP

Previous studies have demonstrated a significant negative correlation between LA reservoir strain and NTproBNP in patients with acute myocardial infarction [31, 32], suspected heart failure [33, 34] and end-stage renal disease on chronic hemodialysis [35] (*r* = -0.41 ~ -0.57). In Kurt's study, LA reservoir strain was more closely related to NTproBNP than LA contractile strain [36]. Unlike the aforementioned studies, LA reservoir and contractile strain were poorly correlated with NTproBNP in our community-based population (*r* = -0.2 ~ -0.3). However, LA contractile strain represented a distinct feature of predicting abnormal NTproBNP in the community population, independent of LAVI. As is known, LA enlargement was found to be an indicator for the severity and duration of increased LV filling pressure [37], and LAVI was positively correlated with plasma BNP levels [38]. Our results propose that LA function by strain might act as an early useful index in the community population before LA enlargement, and a combination of LA strain with LA size might provide more useful information. Future studies might provide new information on LA strain and the cardiovascular outcomes in community populations.

### Role of LASI as a single index

LA stiffness index (LASI), as the ratio of E/e′ to LA reservoir strain, is a new derivative of the LA strain. The ratio of invasively measured PCWP and left atrial systolic strain is used to estimate LA stiffness, representing the change in pressure required to increase the volume of LA. Alternatively, the E/E' ratio is used instead of PCWP in conjunction with the LA strain as a noninvasive measure [20].

LASI [39, 40] or LA compliance (the reciprocal of LASI) [7] is useful in predicting elevated LV filling pressures and identifying patients with HFpEF. In our community-based population, LASI was also correlated with plasma NTproBNP and other echocardiographic parameters demonstrating raised left ventricular filling pressures (E', LAVI, TR velocity). In a study among systemic sclerosis patients, LASI was the best single index in predicting elevated NTproBNP compared with LAVI, LA strain and E/E' [41]. An increased LASI can be used as a marker of early target organ damage in hypertension in a recent paper [24]. In our asymptomatic community–based population, LASI failed to show superiority to conventional measures (LA contractile strain, E/E’ and LAVI) in predicting elevated NTproBNP. As a new index, LASI deserves more attention and further studies.

In our study, images were acquired on GE platform and LA strain indexes were analyzed using EchoPAC software (Version 11.0). Vendor differences are based primarily on strain algorithms. There are also some differences in the strain algorithm among different EchoPAC versions, according to the vendor. In a meta-analysis on speckle-tracking LA strain, the authors found no significant difference between EchoPAC and non-EchoPAC platforms, or difference between different iterations of EchoPAC software [22]. Therefore, we speculated the differences in LA strain indexes between our study and other studies were mainly caused by different pathophysiological states of patients. However, we should remain aware of the potential variations in techniques, and hopefully more comparable strain measurements from different software will be obtained in the future.

To our knowledge, this is one of the few studies to address the association between LA deformation parameters and plasma NTproBNP in asymptomatic communities.

### Limitations

The study was a cross-sectional study with a relatively small sample size and lacked clinical follow-up. There might be a selection bias in the collection of the cohort since limited subjects with LA enlargement were found in this population with a mean age of 66 years, and we excluded those subjects for further analysis. LV strain or SR indexes were not included in our analysis. LA strain and SR indexes were obtained in the apical four-chamber view, while the apical two-chamber view was not included. Several noncardiac presentations affect plasma NTproBNP values, such as ischemic stroke or chronic obstructive pulmonary disease, which might be confounding.

## Conclusions

Our data suggested that LA reservoir and pump functions demonstrated by LA strain and SR were significantly impaired in the community-based population with abnormal plasma NTproBNP levels. LA reservoir strain adds incremental information in predicting abnormal NTproBNP levels to that provided by LA volume and E/E' assessment. LASI, as the ratio of E/e′ to LA reservoir strain, demonstrated similar diagnostic value with LAVI and E/E’ in the detection of abnormal NTproBNP.

## Data Availability

All available data can be obtained by contacting the corresponding author on reasonable request.

## Abbreviations

LA: Left atrium (atrial)
SR: Strain rate
ε_CD_: LA conduit strain, peak positive LA strain during early diastole of the left ventricle
ε_CT_: LA contractile strain, peak negative LA strain during late diastole of the left ventricle (atrial systole)
ε_R_: LA reservoir strain, the sum of ε_CD_ and ε_CT__._
SRe: The first negative peak strain rate during early diastole of the left ventricle, corresponding to LA conduit function
SRa: The second negative peak strain rate during late diastole of the left ventricle (atrial systole), corresponding to LA pump function
SRs: Positive peak strain rate during systole of the left ventricle, corresponding to LA reservoir function
A: Peak velocity during late diastolic of mitral flow
E: Peak velocity during early diastolic of mitral flow
E’: The average of septal and lateral mitral annular early diastolic peak velocity
A’: The average of septal and lateral mitral annular late diastolic peak velocity
VTR: Peak velocity of tricuspid regurgitation
LASI: Left atrial stiffness index
LAVI: Maximal left atrial volume index
LVDd: Left ventricular diastolic diameter
LVMI: Left ventricular mass index
LVEF: Left ventricular ejection fraction
HFpEF: Heart failure with preserved ejection fraction
CAD: Coronary artery disease
BMI: Body mass index
BSA: Body surface area
eGFR: Estimated glomerular filtration rate
ROC: Receiver operating characteristic
LOA: Limits of agreement
